# Chitin Nanofibrils in Poly(Lactic Acid) (PLA) Nanocomposites: Dispersion and Thermo-Mechanical Properties

**DOI:** 10.3390/ijms20030504

**Published:** 2019-01-24

**Authors:** Maria-Beatrice Coltelli, Patrizia Cinelli, Vito Gigante, Laura Aliotta, Pierfrancesco Morganti, Luca Panariello, Andrea Lazzeri

**Affiliations:** 1 Department of Civil and Industrial Engineering, University of Pisa, Via Diotisalvi 2, 56122 Pisa, Italy; patrizia.cinelli@unipi.it (P.C.); vito.gigante@dici.unipi.it (V.G.); laura.aliotta@dici.unipi.it (L.A.); luca.panariello@ing.unipi.it (L.P.); 2 National InterUniversity Consortium of Materials Science and Technology (INSTM), Via Giusti 9, 50121 Florence, Italy; 3 Skin Pharmacology and Dermatology Unit, Campania University “Luigi Vanvitelli”, 80100 Naples, Italy; pierfrancesco.morganti@mavicosmetics.it; 4 MAVI SUD, Aprilia (LT), 04011 Aprilia, Italy

**Keywords:** chitin nanofibrils, poly(lactic acid), nanocomposites

## Abstract

Chitin-nanofibrils are obtained in water suspension at low concentration, as nanoparticles normally are, to avoid their aggregation. The addition of the fibrils in molten PLA during extrusion is thus difficult and disadvantageous. In the present paper, the use of poly(ethylene glycol) (PEG) is proposed to prepare a solid pre-composite by water evaporation. The pre-composite is then added to PLA in the extruder to obtain transparent nanocomposites. The amount of PEG and chitin nanofibrils was varied in the nanocomposites to compare the reinforcement due to nanofibrils and plasticization due to the presence of PEG, as well as for extrapolating, where possible, the properties of reinforcement due to chitin nanofibrils exclusively. Thermal and morphological properties of nanocomposites were also investigated. This study concluded that chitin nanofibrils, added as reinforcing filler up to 12% by weight, do not alter the properties of the PLA based material; hence, this additive can be used in bioplastic items mainly exploiting its intrinsic anti-microbial and skin regenerating properties.

## 1. Introduction

Chitin is the second most abundant biopolymer on earth, having global reserves of 100 billion tons [1]. Waste from the seafood industry is a great source of chitin, because it represents the matrix of the hierarchically structured, fiber-based composite constituting the exoskeleton of crustaceans [2]. On a global level, about 6 mega tons of crustacean shells are discarded per year [3]. For this reason, many researchers evidenced the possibility of exploiting this resource [4] to obtain valuable materials such as chitosan and its derivatives [5], chitin nanofibrils [6,7,8], inorganics such as calcium carbonate, and molecules such as acetic acid and pyrrole [9].

Chitin nanofibrils (CNs), consisting of colloidal nano-rods, represent the crystalline fraction of the chitin extracted from sea food waste; it was reported that they show anti-microbial properties and favor cells regeneration [10,11,12].

Poly(lactic acid) (PLA) is a fully renewable polymer compatible with the human body, plants and the environment, being both resorbable in the human body and biodegradable in composting plants [13]. It is reported to have biocidal activity thanks to its tendency to hydrolyze on the surface, producing lactic acid, which exerts a slight anti-microbial activity [14]. For all these reasons PLA is one of the best alternatives to petrol-based polymers in the packaging, agricultural, personal care, cosmetic, biomedical and tissue engineering sectors [15,16,17]. The modulation of PLA properties to make it suitable for several processing techniques and final applications is an attractive topic of current research about nanomaterials [18]. The possibility of reinforcing PLA using nanofibers was investigated by considering several nano-reinforcements [19] such as cellulose nanocrystals or nanofibers [20,21], sometimes also combined with nanofillers with different aspect ratios, like clays, to modulate barrier properties [22]. Nanocellulose did not show anti-microbial and cell regenerative properties.

In contrast, the combination of PLA and CNs to obtain bionanocomposites can represent a good opportunity for the preparation of bioplastic materials with improved structural and functional properties [23]. Moreover, it is reported that chitin nanofibers require less energy intensive treatments in their production, compared to cellulose nanofibers [24], and that the combination with cellulose nanofibers to obtained nanocomposites can be synergistic [25].

The dispersion of CNs at a nanometric level in PLA was attempted by several techniques. The preliminary modification of chitin nanofibrils was identified as a good strategy to improve its stability in suspension, as observed by Araki, that prepared sterically stabilized chitin nanowhiskers by surface grafting monomethoxy poly(ethylene glycol) (mPEG) [26]. mPEG grafting has been previously reported to effectively improve the dispersion stability of cellulose nanowhiskers [27,28]. By following this strategy based on CNs chemical modification, Zhang et al. [29] acetylated chitin nanofibrils to improve their dispersion in poly (lactic acid) (PLA), but the mechanical properties of composites prepared by solvent casting (not in the molten state) were not improved. Moreover, the chemical modification of diluted suspension can be complex, in view of an industrialization of the composite’s production. On the other hand, extrusion is a conventional melt polymer-processing technique, and it would be preferentially adopted for nanocomposite processing for industrial applications. Rizvi et al. [30], working in a twin-screw laboratory extruder, used more traditional compatibilizing agents to improve the adhesion between nanofibrils and the PLA matrix, investigating the effect of different contents of chitin nano-fibrils in melt-blended PLA/chitin and PLA/CN composites. In particular, maleic anhydride (MA) was grafted to PLA at about 2% by weight, producing a modified polymer (PLA-g-MA). Tensile tests showed an effective increase in Young’s modulus in nano-composites compared to pure PLA. In particular, it was observed that the Young’s modulus underwent an increase of up to 5% chitin content, while higher quantities did not produce any further reinforcement effect. Herrera et al. noticed that only 1% CNs was enough to observe an increase in Young’s Modulus. [31]. The tensile strength decreased by increasing the nano-chitin content, but in any case, lower values than those of pure PLA were observed. This decreasing trend was attributed to the hydrolysis of PLA during the preparation, since aqueous solutions of CNs were added in the melt mixer to avoid agglomeration, and therefore, as the amount of reinforcement used increased, the introduced moisture increased accordingly, leading to an increasing impact of hydrolysis. This paper showed the good potential of CNs to improve mechanical performance, but at the same time, the difficulty of dispersing them at the nano-metric level and homogeneously in the PLA matrix. Guan and Naguib [32] investigated PLA/CN nano-composites using MA as a compatibilizing agent and N,N-dimethylacetamide (DMAc) as a dispersing agent. The latter was added to improve the dispersion of nano-fibrils in the composite. The CNs were re-dispersed in DMAc by mechanical stirring to prepare a nano-structured suspension. Moreover, PLA was prepared with grafted anhydride groups (PLA-g-MA) using 2 wt% of MA and 0.5 wt% peroxide as a radical initiator to improve the fiber-matrix adhesion. Tensile test results demonstrated that the presence of both DMAc and PLA-g-MA, without CNs, led to a decrease of the Young’s modulus and of tensile strength; however, DMAc caused a significant worsening of the mechanical properties, explained by the authors by considering the degradation generated in the PLA through chain scission during the extrusion. It is also important to note that as the amount of nano-filler increased, two contrasting effects occurred: a reinforcing effect of chitin, which increased stiffness and strength at the expense of ductility; and a negative impact on the mechanical properties which was attributable to the presence of DMAc, which caused the degradation of the matrix. This article evidenced the need to use a dispersing agent, looking for one that does not negatively impact the mechanical performance of PLA. The use of known plasticizers of PLA in PLA-based nanocomposites has been studied by Herrera et al. [31,33,34], who developed triethyl citrate (TEC) in water/alcohol suspensions, fed in the extruder, and investigated the properties of composites containing 1% of CNs. They reported that this amount was enough to modify some key properties, such as anti-fungal activity and antistatic behavior puncture strength, and to improve mechanical properties thanks to the very high surface-to-volume ratio of the nanofibrils. Interestingly, the authors investigated the thermal properties of nanocomposites by DSC, and concluded that the simultaneous presence of CNs and TEC in the explored composition range did not provoke significant changes, except for the evident decrease of the glass transition due to the plasticizing effect of TEC. The effect of the plasticizer content to determine the synergic effect of the plasticizer as a dispersing and toughening aid with a minimum impact on the properties of PLA was considered an interesting topic to further investigate by the authors [31]. This molecule, as well as other citrates, due to the presence of ester groups, showed a very high affinity for PLA matrix [35,36,37,38], thus strongly favoring the nanodispersion of CNs in the matrix and the formation of an extended CN/PLA interface.

Poly(ethylene glycol) (PEG) was also successfully used to disperse CNs [39,40,41] in PLA. More recently, Li et al. [42], aiming at obtaining rigid nanocomposites, used high molecular weight poly(ethylene oxide) (PEO) or PEG and investigated the flexural and impact properties of composites with NC in the range 10–40%, observing a reinforcing effect of CN.

In all these studies, the exigence of effectively dispersing the CNs in PLA led to the necessity of using both a plasticizer and chitin nanofibrils.

Interestingly, Nakagaito et al. [43] used only water as a dispersion medium, which was removed by filtration and drying. Although PLA is insoluble in water, aqueous suspensions can be obtained by using PLA short fibers or particles, that can be easily mixed with cellulose nanofibers in water suspension. After dewatering, the mixture forms paper-like sheets that can be laminated and compression molded. Hence, nanocomposites were obtained by compression molding the filtrates. Static tensile test and dynamic mechanical analysis were performed to evaluate the reinforcement as a function of nanofiber content. Chitin nanofibers delivered reinforcement similar to that of cellulose nanofibers, being especially effective at up to 70 wt% fiber load. The ultimate tensile modulus and strength reached 7.7 GPa and 110 MPa, respectively, at a nanofiber content of 70 wt%. This work evidenced the interesting potential of CNs as a reinforcing agent in a pure PLA matrix. This methodology, however, cannot claim to reach full nanoscaled homogeneity because of the anisotropy flows typical of compression molding process. Subsequently, Li [42] compared PEG and PEO dispersion with this methodology, using a laboratory twin screw extruder. In this case, the Modulus measured by DMTA resultedin 7.6 GPa for the water method (a value in agreement with the one reported by Nakagaito et al. [43]), and 6,5 and 6,0 GPa for PEG and PEO respectively. The lower value obtained by using PEG and PEO can be attributed to their plasticizing effect.

Interestingly, very recently, Shanshina et al. [44] used an ionic liquid-based approach to co-dissolve PLA and CNs and produce PLA fibers containing up to 27% by weight of CNs. The fibers showed improved strength with respect to the fibers obtained by pure PLA. However, processes considering ionic liquid technology, which may be promising for future applications in several fields, are not yet well diffused in the industry.

By an analysis of the literature, it is evident that the combination of reinforcement with the necessity of using dispersing agents is an interesting topic of current research. The use of PEG resulted an effective overall limit of CN agglomeration, but a systematic study about the possibility of modulating nanocomposite properties, considering low amount of CNs, was never carried out. Hence, in this attempt, after the preparation of nanocomposites, their tensile and thermal properties as a function of the content of CNs and PEG were measured and discussed.

## 2. Results

### 2.1. Dispersion of Chitin Nanofibrils in Plasticized Pla

**Figure 1:**
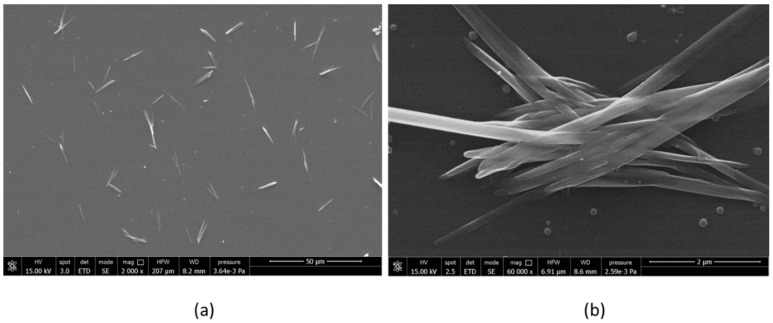
FESEM (field emission scanning electron microscopy) micrographs obtained from 2% by weight water suspension of CN diluted 1:100 and deposited on glass. (**a**) 2000× magnification; (**b**) 60,000× magnification.

In order to study chitin nanofibrils morphology, water suspensions at 2% by weight of chitin nanofibrils were diluted 1:100, and then one drop of diluted suspension was deposited onto a glass window. By using a field emission scanning electron microscope (FESEM), it was possible to determine the shape of chitin nanofibrils (Figure 1): average length of 20 µm and an average width of 90 nm.

**Figure 2:**
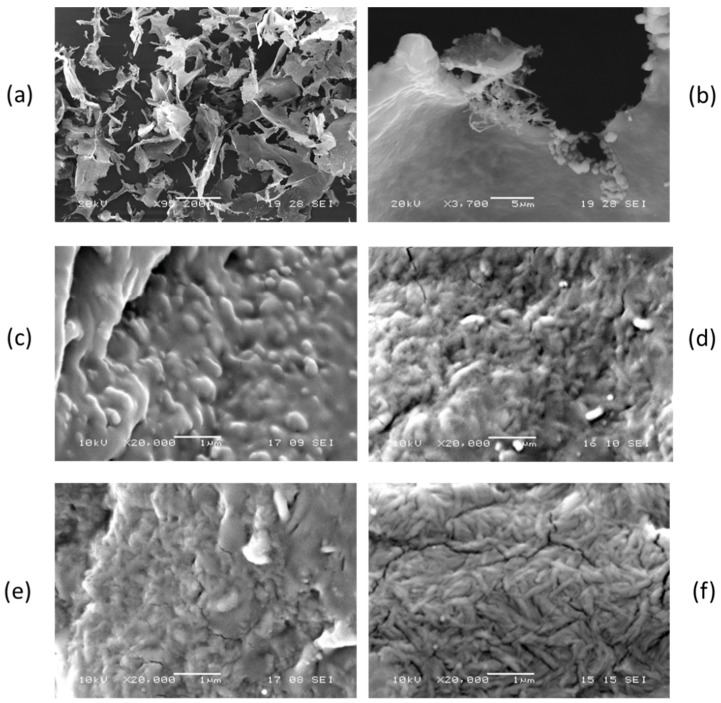
SEM micrographs of (**a**) dried CN suspension flakes; (**b**) magnification of flake edge; (**c**) pre-composite based on PEG 1500; (**d**) pre-composite based on PEG 4000; (**e**) pre-composite based on PEG 6000; (**f**) pre-composite based on PEG 8000.

When the 2% by weight suspension was dried without any previous dilution, and characterized by Scanning Electron Microscopy (SEM), the chitin nanofibrils formed agglomerates (Figure 2). In Figure 2a, it is evident that the drying of nanosized chitin produced large flakes in which the fibers are agglomerated to form a compact structure like that of a sheet of paper. In fact, in between different nanofibrils, having a very high surface to volume ratio, the formation of a high number of hydrogen bonds is thermodynamically favored, and this reasonably results in this compact structure. The structure of a nano-fibrous disordered assembly is clearly observable at the edges of the flakes (Figure 2b).

With a concentration of 1:10 of the CN suspension, PEGs having 400, 1500, 4000, 6000 and 8000 as molecular weight were added in weight ratio 1:1 to CN to obtain five different pre-composites that were dried. The obtained materials were solid, except for the one obtained with PEG 400, that showed a pasty consistency. The solid pre-composites were characterized by SEM and showed, from a morphological point of view, a complex fibrous nano-structure (Figure 2c–f), indicating that PEG constituted the matrix in which chitin nano-fibrils were immersed. Hence, it seemed reasonable that a PEG polymer was present in between the CNs, thus avoiding the formation of compact agglomerates.

With the purpose of investigating the interactions between PEG and chitin nanofibrils, some specific infrared characterizations and thermogravimetric tests were performed on the sample obtained by adding to chitin nanofibrils 2% of PEG 8000. The highest molecular weight was selected because, with respect to the same quantity of lower molecular weight PEG, it corresponded to a lower number of macromolecules, and consequently, demonstrated less efficient interactions with the CN. Thus, if the PEG 8000 is able to interact with the CNs, this effect will be stronger in samples with a lower molecular weight.

**Figure 3:**
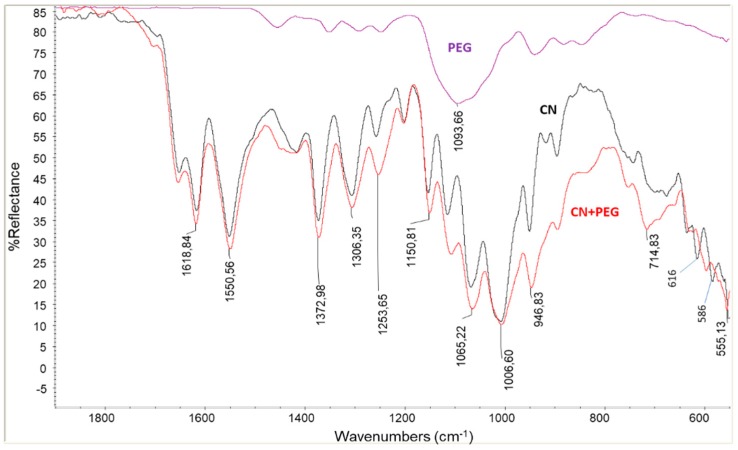
infrared ATR spectra of PEG 8000, CN and mixture CN/PEG8000 98:2. The spectrum of pure PEG is reported with a reduced reflectance intensity to allow a better visualization of the spectra.

Regarding the infrared spectrum, CN showed characteristic amide I and Amide II bands at 1618 and 1550 cm^−1^ respectively. The Amide I band is typical of α-chitin [45], as it is split into two components at 1660 and 1630 cm^−1^. This double band was attributed to the influence of hydrogen bonding or the presence of an enol form of the amide moiety [46,47,48]). Interestingly, it was found that the infrared bands typical of chitin resulted in the spectrum of the sample containing PEG8000, despite of only 2% of PEG8000 being present. This is particularly evident in the spectrum part where PEG 8000 (Figure 3) did not show any absorption bands, such as in the region 500–800 cm^−1^ and in the region 1500–1800 cm^−1^. The presence of PEG, that probably at least partially interposes between CNs, thus favors the hypothesis of more complex interactions in between CNs.

**Figure 4:**
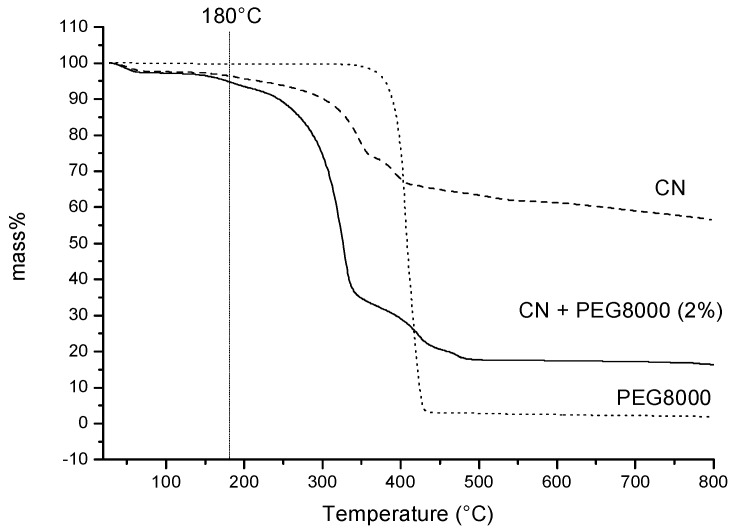
thermogravimetric curves of CN, PEG and CN + PEG (2%). The adopted extrusion temperature is marked with a dash line.

The thermo-gravimetric analysis of pure CN in nitrogen flow showed that its main degradation step is at 349 °C (−21.2% by weight), and a second evident mass loss at 394 °C (−9.5%) is present. These values were calculated through the analysis of the derivative curve of the thermogram. A slight loss of mass can be observed also below 140 °C, but it accounts for a reduced loss of mass of the pure chitin (−2.6%). The mass loss observed below 100 °C was attributed to removal of humidity (−4%). The final residue was 58.1%. In the presence of 2% of PEG the thermogravimetry trend was completely changed (Figure 4). The main mass loss at 329 °C was −57.5%, and the final residue presented as 17.4% by weight. Hence, the presence of PEG induced a more efficient thermal degradation of CN, probably because it interposes between the CNs, avoiding the formation of compact agglomerations. This type of assembly is instead typical of pure CN, where inter-fibrils hydrogen bonding is predominant, resulting in the formation of a considerable quantity of carbonaceous residue after thermal degradation in nitrogen atmosphere. Similar results were obtained by Cheng et al., that considered the addition of PEG 1000 to cellulose nanofibrils [49].

As the morphology of the different composites was very similar, only PEG400 liquid and PEG8000 solid (having the lowest and highest molecular weights respectively) -based samples were considered for preparing PLA based composites in a mini-extruder. The composition of the different composites, reported in Table 1, was selected by considering not only the two different molecular weights, but also with the aim of investigating the composite’s properties as a function of PEG concentration and chitin nanofibrils concentration. As in the pre-composite, the weight ratio of PEG and CN was 1:1; for this reason, some PEG was added to obtain the desired content. For the P1Low2NC, a specific pre-composite was prepared with a PEG: CN weight ratio of 1:2.

**Figure 5:**
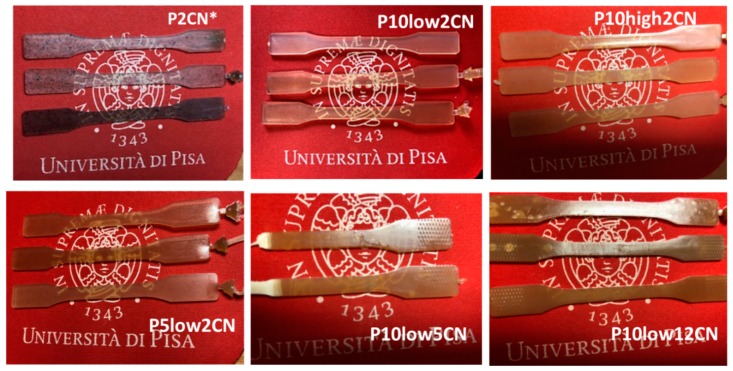
specimens obtained by injection moulding: comparison regarding transparency.

The extrusion was followed by the injection molding of specimens, that resulted in a transparent com pound, which was in agreement with the achievement of a nano-scaled dispersion [50,51]. As shown in Figure 5, P10low2CN and P10high 2CN resulted in a transparent compound, and also P5low2CN, but with the presence of some visible particles.

P10low5CN resulted in a transparent but brownish compound, whereas the sample containing 12% of CN (P10low12CN), resulted in a brown compound with a reduced transparency. In general, high transparency and colorlessness was achieved by decreasing the content of CN and increasing the content of PEG. In fact, both these characteristics positively affect the CN nano dispersion. The role of PEG as a dispersing agent is evident. In fact, the P2CN* composite was prepared by adding dried chitin nanofibrils without using PEG, and in this case, the resulting specimens were darker and not transparent because of the presence of visible particles. In this case, the CN formed aggregates that could not be well dispersed in the melt PLA during extrusion.

**Figure 6:**
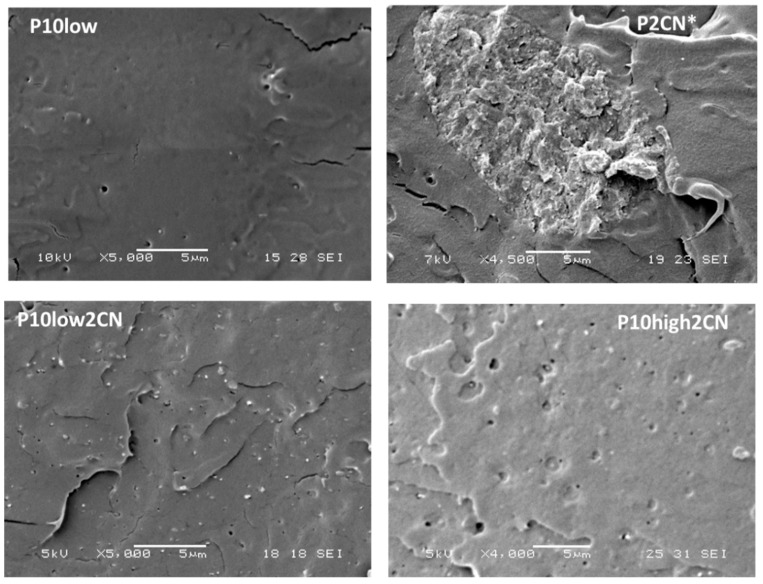
SEM micrographs obtained on cryofractured surfaces obtained from tensile specimens of different PLA/Chitin nanofibrils composites.

The samples were then characterized by SEM microscopy by preparing cryofractured surfaces from tensile specimens. From the micrographs in Figure 6, it is possible to see that the PLA containing only PEG400 (P10low sample) present a very high homogeneity apart from the presence of some round domains attributable to the presence of a second phase of PEG. The presence of very big aggregates, i.e., with diameters higher than 10 microns, could be observed in the sample obtained without preparing a pre-composite (P2CN*). In other composites where the chitin nanofibrils were dispersed by the addition of the pre-composite, it was not possible to reveal the presence of chitin nanofibrils aggregates, although several cryofractured surfaces were examined. The micrographs results were similar to those of the sample obtained in absence chitin nanofibrils, with submicrometric droplets being attributable to PEG. This result indicated a very good dispersion of chitin nanofibrils in the composite.

**Figure 7:**
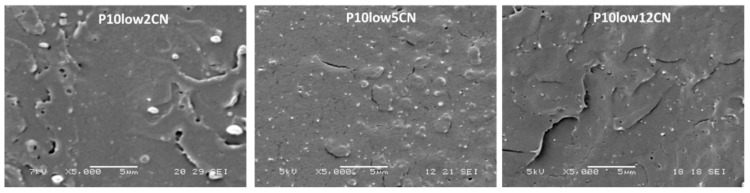
SEM micrographs obtained on cryofractured surfaces obtained from tensile specimens of different PLA/Chitin nanofibrils composites. From left to right the content of CN increases.

Interestingly, it was found that the dimensions of the PEG domains seemed to decrease by increasing the content of CN (Figure 7), reasonably indicating not negligible interactions between chitin nanofibrils and PEG 400, leading to a better dispersion of PEG. The interactions may be responsible for this decrease, considering that chitin nanofibrils are present both in the plasticized PLA matrix and in the PEG domains, thus acting as interfacial stabilizers. Moreover, micrometric PEG-based domains consist, during the melt extrusion of PLA nanocomposites, of liquid pools with a viscosity lower than that of the matrix, and these pools can contain CNs. The viscosity of such a liquid is increased because of the presence of chitin nanofibrils. In fact, in general, the viscosity of a suspension increases as a function of nanofibrils content [52,53], because of the tendency of gelling of the nanofibrils. The dimension of domains in immiscible polymer blends is reported to be dependent on the viscosity ratio, and in general it decreases when the ratio of the viscosity of the matrix and the dispersed phase is close to 1 [54,55]. The decrease of the PEG domain dimensions can be thus additionally ascribed to the viscosity variations in the two phases due to the presence in both of PEG and chitin nanofibrils.

**Figure 8:**
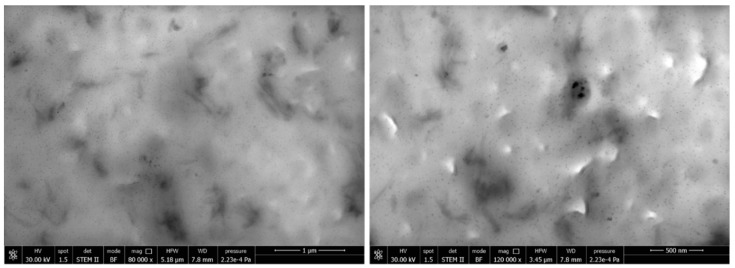
TEM micrographs obtained on the P10low2CN sample at different magnifications.

The composites P10low2CN was characterized by STEM microscopy (Figure 8) that revealed the presence of CNs as single fibers and bundles, dispersed at the nanometric scale. A good correspondence with the dimensions observed in FESEM micrographs of CNs (Figure 1) is evident.

**Figure 9:**
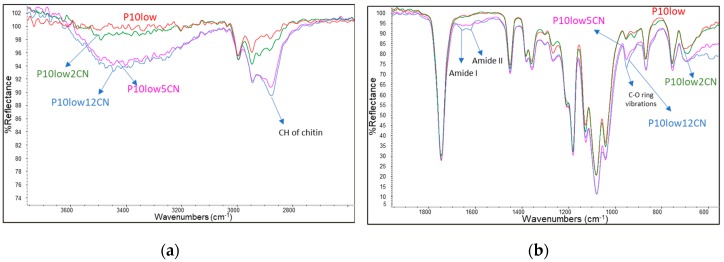
(**a**,**b**) Infrared ATR spectra of P10low, P10low2CN, P10low5CN and P10low12CN.

Infrared ATR spectroscopy was applied (Figure 9) to investigate the distribution of chitin nanofibrils on the surface of injection molded specimens. The ATR technique allowed us to record the vibrational spectrum of a material present on a surface at up to a few microns. In particular, the Smart itX ATR diamond plate allows a depth of penetration 2.03 micrometers at 1000 cm^−1^. By overlaying the spectra of P10low, as a reference, with the spectra of the samples containing increasing amounts of CNs (2%, 5% and 12% by weight), it was found that some bands attributable to CNs are revealed. These bands are extremely weak in P10low2CN sample, whereas they are well evident in P10low5CN and P10low12CN. It is thus evident that a significant number of CNs are present on the specimen surface when the content of CNs is above 5% by weight. If the content is lower, the presence of CNs can not be revealed by this technique.

### 2.2. Tensile Properties

Tensile tests were performed on specimens produced by injection molding and conditioned for two weeks at 50% as relative humidity. The pure PLA is brittle and shows a high value of Young’s Modulus (3.5 GPa), a high value of stress at break, but a low value of elongation at break (Table 2). When the PLA is plasticized using PEG 400 (trial P10low), a strong decrease in Young’s Modulus and stress at break and an increase in elongation at break (up to 180%) were observed, in agreement with an increased ductility of the material. Interestingly, the addition of CN to plasticized PLA resulted in a decrease in Young’s Modulus and stress at break, whereas the elongation at break was not significantly affected by the presence of CNs, also when their content was increased in the composites. Interestingly, the addition of PEG 400 and PEG 8000 resulted in similar properties, with the latter composite showing a higher Young’s Modulus and stress at break than the former.

### 2.3. Thermal Properties

Thermal properties were recorded on nanocomposite injection molded specimens after a few months from preparation following a methodology consisting of a first heating step, cooling, and a second heating. Regarding the first heating (Table 3), it showed a glass transition T_g_ with an evident peak of enthalpy relaxation due to the ordering of PLA chains during the specimen storing period. The presence of PEG determined the decrease in the glass transition temperature and the decrease of the cold crystallization temperature. The cold crystallization temperature T_cc_ did not significantly change as a function of CN content, whereas the crystallinity significantly changed (Table 3).

Regarding the second heating (Table 4), it is evident that the cold crystallization temperature significantly decreased as a function of CN content, in agreement with a slight nucleating action of the CNs. This effect is evident only in the second heating thanks to the lower content of crystallinity X_c_ developed during the controlled cooling step.

## 3. Discussion

The obtained results regarding phase morphology characterization, as well as the analysis of properties, agree with the achievement of a nanoscaled dispersion of chitin nanofibrils in plasticized PLA. The SEM characterization (Figure 6 and Figure 7) and the observation of the optical properties of the injection molded specimens (Figure 5) evidenced this achievement and showed that it is dependent on the CN content and PEG content in the composites.

Thanks to infrared ATR characterization analysis, it was possible to show the clear presence of the CN bands on the injection molded specimens surface in samples containing more than 5% by weight of CN. Conditions for dispersing CNs in the bulk that resulted in the effective presence of CNs on the surface of injection molded specimens, for potentially exploiting their functional anti-microbial properties, were thus evidenced. It is interesting to note that, in the case where injection molded products are in a hot and humid environment (e.g., in applications related to human body, like implants or surgical suture wires), the surface of the PLA can degrade by hydrolysis, leaving the chitin nanofibrils to emerge on the surface. This behavior allows the preservation over time of the functional characteristics of CNs on the surface, even in the case of slow degradation of the PLA.

**Figure 10:**
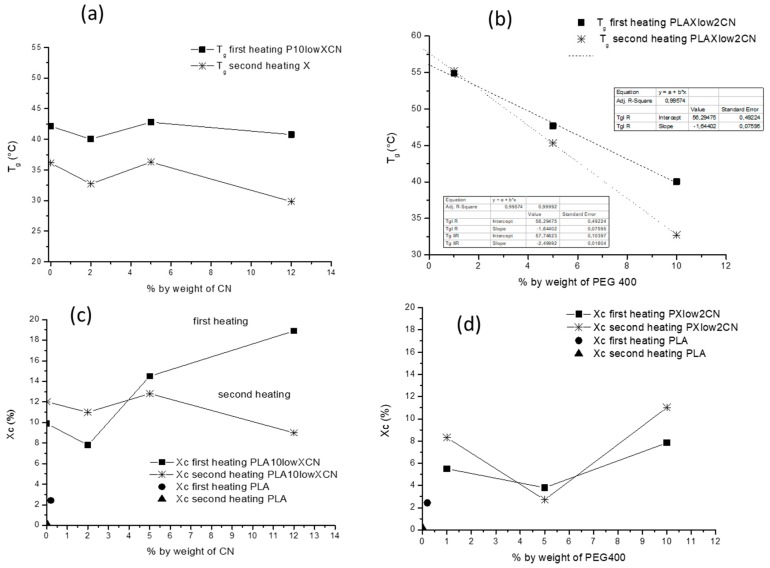
Analysis of thermal properties from first and second heating steps in PLA/Cn nanocomposites: (**a**) trend of Tg as a function of % by weight of CN; (**b**) trend of Tg as a function of PEG 400 content and dashed lines to extrapolate Tg at 0% by weight of PEG; (**c**) trend of crystallinity as function of % by weight of CN; (**d**) trend of crystallinity as a function of % by weight of PEG 400.

The thermal properties of the nanocomposites were analyzed as a function of CN and PEG 400 content (Figure 10). The trend of the glass transition as a function of the CN content is reported in Figure 10a. The values are almost constant but higher for the first heating than for the second. This difference can be ascribed to the formation of ordered structure in the sample injection molded and stored for some months (first heating), and is also responsible of the presence of the enthalpy relaxation peak in the glass transition range. Interestingly the glass transition temperature (T_g_) showed an almost linear trend both in the first and second heating as a function of the PEG content (Figure 10b). The highest slope for the second heating trend can be explained by considering that in the first heating, the enthalpy relation made the samples less sensitive to plasticizer content. The linear fitting of the two trends allowed us to determine the intercept value of the line, corresponding to the glass transition temperature of nanocomposites without PEG. The values of extrapolated T_g_s for pure PLA containing 2% of CN of 56.3 and 57.7 °C for first and second heating respectively. Interestingly the values recorded for the P2CN* sample were 57,5 and 55,2 °C for the first and second heatings, respectively (Table 3 and Table 4). In the first heating, due to the uncontrolled storing conditions, the difference of about 1 °C seems to be irrelevant. In the second heating, recorded after a controlled cooling step, the increase of 2.5 °C is significant, and can be ascribed to the better dispersion achieved in the sample obtained by dispersing CN by using PEG, resulting in a better interaction between CNs and PLA matrix, than in the sample P2CN*, where CNs were present in agglomerated micrometric particles (Figure 6).

The crystallinity X_c_ was almost constant as a function of CN content in the second heating, showing an insignificant effect of CNs on controlled crystallization. In contrast, the crystallinity was significantly increased when the content of CN was 5% and 12% by weight. As the cooling of the injection molding process was very fast, this difference can be ascribed to the crystallinity developed during the storing of specimens. Hence, the presence of CNs in amounts higher than 2% resulted in an increase of crystallinity in the injection molding specimens during the storage that resulted in shrinkage or slow plasticizer expulsion. The surface of P10low5CN and P10low12CN became oily after some months from their preparation, whereas the P10low2CN specimens did not show any surface oiliness. This evidence may be relevant in view of the application of these nanocomposites to the injection molding sector.

The crystallinity as a function of % by weight of PEG400 for the nanocomposites at 2% by weight of CN showed a minimum value for both first and second heating at 5% by weight of PEG. The trend is like the one observed for plasticized and nucleated PLA by Fehri et al. [35]. When the concentration of the plasticizer is low (up to 5%), it hinders the crystal growth with respect to pure PLA. In contrast, when the concentration is high, the main effect of the plasticizer is to provide a higher free volume for segments motions, allowing the chain fragments to assemble more easily in crystals.

Based on the slight changes in properties observed in cases of relatively fast cooling conditions, the effect of thermal properties on tensile properties, performed a few days after the preparation of specimens by injection molding, can be considered almost negligible, in agreement with the studies carried out by Herrera et al. [31].

**Figure 11:**
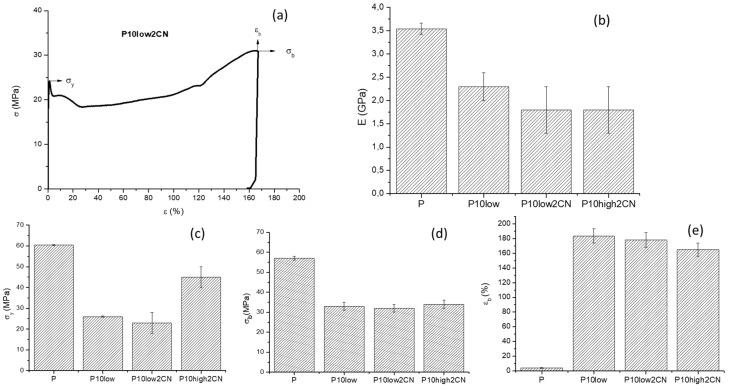
Tensile results to compare the use of PEG with high and low molecular weight: (**a**) example of stress vs. strain curve of the ductile composites; (**b**) Young’s Modulus E; (**c**) Stress at yield σ_y_; (**d**) stress at break σ_b_; (**e**) elongation at break ε_b_. Standard deviation is reported as error bars.

The stress strain curves recorded for the nanocomposites showed a trend like the one reported in Figure 10a as an example, where the stress at break resulted higher than the stress at yield. In Figure 11, a comparison between PEG 400 and PEG8000 regarding the tensile properties of the samples is proposed. The Young’s Modulus (Figure 11b) decreased by adding the plasticizer alone, but the addition of CN resulted in a further decrease. Hence, the CNs, in the presence of 10% by weight of plasticizer, did not show a clear reinforcing action; this was also the case using PEG400 and PEG8000. The elongation at break (Figure 11e) increased by up to 180% by adding PEG 400 to PLA, and the presence of 2% by weight of nano-dispersed CNs did not result in a decrease in elongation at break both for PEG400 and PEG8000. The stress at break resulted in similar behavior for nanocomposites obtained with PEG400 and PEG8000, whereas the stress at yield resulted higher for PEG8000 than for PEG400. This can be ascribed to the higher mobility allowed in the system by the PEG400, with the lower molecular weight, allowing for easier sliding of macromolecules in correspondence with the beginning of the yield.

The tensile properties were also investigated by considering the trends of the different measurements as a function of chitin nanofibrils content and PEG content.

**Figure 12:**
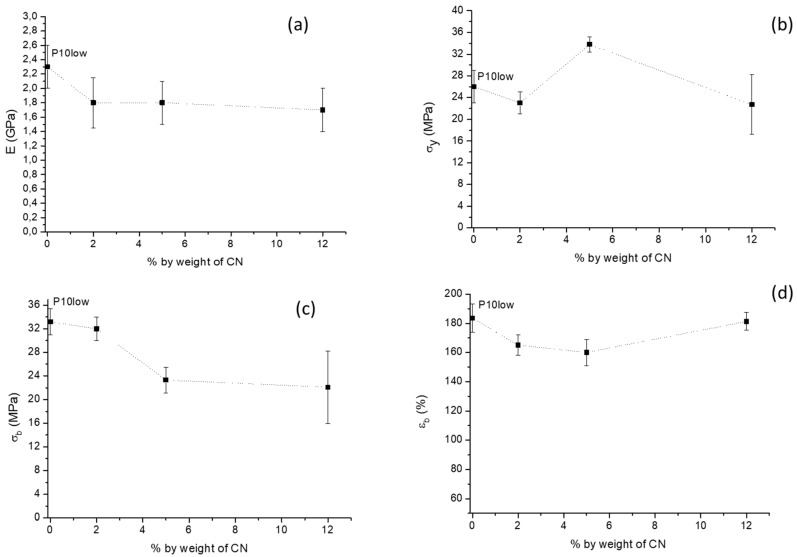
Tensile results as a function of CN content at constant PEG 400 concentration (10% by weight): (**a**) Young’s Modulus E; (**b**) Stress at yield σ_y_; (**c**) stress at break σ_b_; (**d**) elongation at break ε_b_.

Regarding the chitin nanofibrils content investigated in the composite at 10% by weight of PEG400, the elastic Modulus slightly decreased by adding 2% by weight of CN, and remained almost constant at up to 12% by weight (Figure 12a). Stress at yield showed a maximum for the composite at 5% by weight of CN and the stress at break decreased as a function of CN content, showing the highest decrease, i.e., between 2% and 5% by weight, of CN. The nanocomposite at 5% seemed to be the most rigid; this was confirmed by the elongation at break data, showing a minimum for this composite. The variations of elongation at break are much limited in any case, which is in agreement with the good dispersion resulting from morphologic analysis.

**Figure 13:**
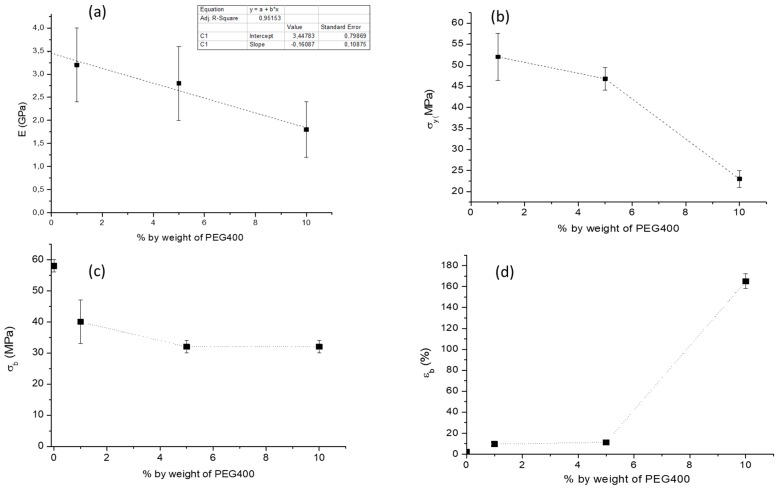
Tensile results as a function of PEG400 content at constant CN concentration (2% by weight): (**a**) Young’s Modulus E; (**b**) Stress at yield σ_y_; (**c**) stress at break σ_b_; (**d**) elongation at break ε_b_.

The stress at yield significantly decreased as a function of PEG content (Figure 13b) because of the increased mobility due to plasticization contributing to decrease the energy required for the sliding of macromolecules with respect to the other. The stress at break slightly decreased as a function of PEG content because of a plasticization effect, decreasing the energy necessary for sample deformation. The elongation at break largely increased in between 5% and 10% by weight of PEG (Figure 13c). Only the nanocomposites obtained with a PEG content of 10% could reach elongation at break values above 150%. The Young’s Modulus as a function of PEG content showed a decreasing linear trend (Figure 13a). The linear fitting allowed us to determine the value of the Modulus for the composite consisting of PLA and CN (Figure 13a) as the intercept of the obtained line. The obtained value by this methodology was 3.45 GPa, representing the Modulus of the PLA/CN 2% nanocomposite. It should be noted that the value obtained for the P2CN* sample presenting micrometric agglomerates of CN (Figure 6) was 2.9 GPa. The increase of 0,55 GPa can be reasonably attributed to the improved dispersion in the PLA matrix achieved thanks to the use of PEG 400. The increased interfacial surface between CN and PLA, favoring matrix-filler interactions, accounts for this improvement. It is important to note that the obtained value results were lower than the value determined for pure PLA (3.5 GPa). Hence the CNs could not reinforce the PLA matrix, either when micrometrically dispersed (P2CN*) or when nanometrically dispersed (extrapolated intercept value). These results are different from those obtained by Herrera et al. [31], who noticed an increase of Young’s Modulus when only 1% of CNs was added. This effect on the mechanical properties can be attributed to the low affinity of the dispersing agent for the PLA matrix, TEC, used by these authors. This molecule, as well as other citrates, due to the presence of ester groups, showed a very high affinity for the PLA matrix [35,36,37], thus strongly favoring the nanodispersion of CN in the matrix and the formation of an extended CN/PLA interface. In contrast, PEG, having a high affinity for the polar groups of chitin, can support its nanodispersion, but can be more difficultly removed from CNs, remaining in between the CN surface and the PLA matrix. The PEG can thus coat the CN surface, lowering the reinforcing effect of nanofibers.

In literature, some samples prepared by the PEG method were studied, and it was noticed that some short fiber clusters are entangled with PLA matrix with a not evident phase separation between CNs and PLA. This observation revealed that PEG is a good interfacial compatibilizer for CNs and PLA. However, this reinforcing effect may be weakened due to a decrement of the CN aspect ratio. These aspects would require further research to better understand the effect of structural and morphologic parameters on the final properties.

## 4. Materials and Methods

### 4.1. Materials

Chitin nanofibrils (CN) water suspension at a concentration of 2% wt. was produced by MAVI SUD through its patented process [56], starting from chitin coming from seafood waste. For the preparation of the pre-composites, it was concentrated at 20% by weight.

Poly(ethylene glycol) (PEG), a liquid having a molecular weight of 400 (low), and PEG, a solid having a molecular weight of 1500, 4000, 6000 and 8000 (high), were purchased from Aldrich and used without any further purification.

PLA Ingeo™ 2003D, Extrusion Grade with density of 1.24 g/cm^3^, a melt index of 6 g/10 min at 210 °C and 2.16 Kg, produced by NatureWorks LLC. It has a molecular weight of 170,000 g/mol and contains up to 4.1% isomeric D units. It was dried in ventilated oven at 60 °C for 16 h before extrusion trials.

### 4.2. Materials Preparation

PEG400 (or PEG800) were added to concentrated chitin nanofibrils suspension and stirred for two hours at room temperature. The amount was calculated considering that in the final pre-composites, the weight ratio of CN and PEG was 1:1. The obtained semiliquid emulsion was dried in a ventilated oven at 50 °C up to constant weight to obtain a solid when PEG8000, PEG6000, PEG4000 and PEG1500 were used, and a sample with liquid highly viscous consistency when PEG 400 was used.

The extrusion of the PLA 2003D (Ingeo™ Nature Works, Minnetonka, MN, USA) in the presence of PEG8000 or PEG400 was carried out after drying the material for 16 h at 60 °C in a ventilated oven, using a TwinLab II Haake™ Rheomex CTW 5 laboratory screw extruder (Vreden, Germany). After homogenizing using mortar and pestle, the materials were fed into the extruder from the hopper at the beginning of the twin screws, and were mixed into the recirculating channel of the extruder. The extrusion was carried out at 180 °C and 90 rpm for one minute. After extrusion, the molten material was transferred through a preheated cylinder into the Haake™ MiniJet II mini injection molding machine to obtain Haake type III test specimens for tensile testing. The injection molding was carried out at 180 °C, 650 bars, holding time of 15 s, mold temperature of 35 °C.

### 4.3. Characterizations

The analysis of the average length and width of chitin nanofibrils was carried out using ImageJ software applied on micrographs obtained by using a FEI Quanta 450 ESEM FEG field emission instrument.

The morphology of master batches and composites was studied by scanning electron microscopy (SEM) using a JEOL JSM-5600LV instrument and analyzing cryo-fractured surfaces, previously subjected to sputtering with gold.

Infrared spectra were recorded in the 550–4000 cm^−1^ range with a Nicolet 380 Thermo Corporation Fourier Transform Infrared (FTIR) Spectrometer equipped with smart Itx ATR accessory, collecting 256 scans at 4 cm^−1^ resolution

Thermogravimetric analysis was performed on 10–20 mg of sample using a Mettler-Toledo TGA/SDTA 851 instrument (Columbus, OH, USA) operating with nitrogen as the purge gas (60 mL/min) at 10 °C·min^−1^ heating rate in the 25–800 °C temperature range.

The nanocomposites samples were cut with a Reichert Ultracut E ultramicrotome into ultrafine sheet (<1 micron thickness) and collected onto double folding copper grids (50/100 mesh). The grids were closed and coated with a thin layer of carbon by a EMITECH K950 Evaporator Coater Sputter (Laughton, UK) to make them electrically conductive. The micrographs were obtained with a FEI Quanta 450 ESEM FEG in STEM mode.

Tensile tests (UNI EN ISO527) were carried out using a universal INSTRON 5500R test machine with a 1 kN load cell at a speed of 10 mm/min onto specimens conditioned for 2 weeks at 25 °C and relative humidity of 50%.

Differential scanning calorimetry analyzes (DSC) were performed on material sampled from injection molded specimens using a TA Q200 instrument with nitrogen as carrier gas and indium as a calibration standard. The samples were heated from −100 °C to 250 °C at 10 °C/min and cooled from 250 °C to −100 °C at 20 °C/min. The second heating was carried out by heating analogously from −100 °C to 200 °C at 10 °C/min. The crystallinity was calculated by the formula Xc = [(ΔH_m_−ΔH_cc_)/(ΔH_0_·w)] × 100, ΔH_m_ is the melting enthalpy, ΔH_cc_ is the cold crystallization enthalpy, ΔH_0_ is the melting enthalpy of PLA fully crystalline (the value of 93J/g was considered [52]) and w is the weight fraction of PLA in the composite.

## 5. Conclusions

In the present paper, the preparation by extrusion of nanocomposites consisting of a PLA matrix and a dispersed chitin nanofibrils (CN) phase was obtained using a methodology based on the preliminary preparation of pre-composites based on CN and poly(ethylene glycol) (PEG) having a molecular weight of 8000 or 400. The presence of PEG, making it possible to keep the CNs separated, avoiding the problem of their agglomeration due to inter-macromolecular interactions.

The pre-composites were dispersed in molten PLA to obtain nanocomposites with different content of PEG and CN in a laboratory extruder. The morphologic analysis by SEM demonstrated the absence of agglomerated CNs in the sample, in agreement with the presence of a nanostructured material.

The content of PEG and CN strongly influenced the colorlessness and the transparency of the injection molded specimens. In particular, a higher PEG content and a lower CN content resulted in more transparent and colorless specimens. The presence of CNs on the surface of the injection molded specimens was detected in the nanocomposites with a content of CN higher than 5%.

The thermal properties showed few relevant changes due to the addition of CNs to the samples, being only responsible of a slight nucleating action. Interestingly, it was found that in injection molded specimens, the polymeric chains slowly reorganize into ordered structures during storage at room temperature, thus showing both enthalpy relaxation peak and increased crystallinity. This reorganization is evident above 5% by weight of CNs.

The tensile properties did not show a reinforcing effect of CNs, but the achieved good dispersion allowed us to maintain high values of elongation at break (>150%), typical of plasticized PLA, up to 12% by weight of CN. The absence of a reinforcing effect, not in agreement with literature data, was tentatively explained by considering that PEG, having a high affinity for the polar groups of chitin, can support its nano-dispersion, but can be more difficult to remove from CNs, remaining in between CNs surface and PLA matrix. The PEG can thus coat the CN surface, lowering the reinforcing effect of nanofibers. This aspect, useful for selecting the correct CN/dispersing agent system, would necessitate further elucidation.

The methodology investigated and developed here, in comparison with the other methodologies for dispersing CNs in PLA, can offer the advantage of being easily applicable also at an industrial scale, and does not modify the thermo-mechanical properties typical of plasticized PLA. Moreover, this methodology can be advantageous in the case of the study of ductile materials for injection molding or flexible materials, to be applied to plastic films, both with low contents of CNs, in order to exploit their functional properties.

## Figures and Tables

**Table 1:** Composition of composites obtained by PLA, chitin nano-fibrils and PEG.

Samples	PEG (%)	MW of PEG	CN
P	−	−	−
P2CN* ^a^	−	−	2
P10low	10	400	0
P10low2CN	10	400	2
P10high2CN	10	8000	2
P1low2CN	1	400	2
P5low2CN	5	400	2
P10low5CN	10	400	5
P10low12CN	10	400	12

^a^ P2CN* is a sample of Chitin nanofibrils powder obtained by simply drying the CN suspension, without using PEG.

**Table 2:** Tensile properties of the PLA nanocomposites: E is the Young’s Modulus, σ_y_ is the stress at yield, σ_b_ is the stress at break and ε_b_ is the elongation at break.

Samples	E (GPa)	σ_y_ (MPa)	σ_b_ (MPa)	ε_b_ (%)
P	3.5 ± 0.1	60.4 ± 0.3	57 ± 1	4.1± 0.5
P2CN* ^a^	2.9 ± 0.1	−	58 ± 2	2.3 ± 0.4
P10low	2.3 ± 0.3	26 ± 0.3	33 ± 2	180± 10
P10low2CN	1.8 ± 0.3	23 ± 5	32 ± 2	160 ± 10
P10high2CN	2.5 ± 0.1	45 ± 5	34 ± 2	160 ± 10
P1low2CN	3.2 ± 0.8	52 ± 6	40 ± 7	10 ± 2
P5low2CN	2.8 ± 0.8	47 ± 3	32 ± 2	11.4 ± 0.9
P10low5CN	1.8 ± 0.3	34 ± 2	23 ± 2	160 ± 10
P10low12CN	1.7 ± 0.3	23 ± 5	22 ± 6	181 ± 6

^a^ CN* is a sample of Chitin nanofibrils powder obtained by simply drying the CN suspension, without using PEG.

**Table 3:** DSC results related to chitin nanofibrils PLA based nanocomposites (first heating).

	T_g_ (°C)	T_cc_ (°C)	ΔH_cc_ (J/g)	T_m_ (°C)	ΔH_m_ (J/g)	X_c_ (%)
P	57.7	106.9	21.24	149.2/157.4	21.4	0.2
P2CN*	57.5	107.7	23.21	148.8/155.9	25.5	2.4
P10low	42.1	77.6	17.8	154.7	27.1	9.9
P10low2CN	40.0	74.4	18.3	152.1	26.6	7.8
P10high2CN	43.8	76.6	6.7	154.7	33.3	28.4
P1low2CN	54.9	100.9	18.6	147.9/157.9	23.7	5.5
P5low2CN	47.7	88.6	15.6	(142.3)/155.9	19.2	3.8
P10low5CN	42.8	74.1	15.5	154.2	29.0	14.5
P10low12CN	40.8	75.5	11.4	152.3	29.1	19.0

T_g_ = glass transition temperature; Tcc = crystallization during heating peak temperature; ΔHcc = enthalpy of crystallization during heating; Tm = melting peak temperature; ΔHm = integral of the melting peak.

**Table 4:** DSC results related to chitin nanofibrils PLA based nanocomposites (second heating).

	T_g_ (°C)	T_cc_ (°C)	ΔH_cc_ (J/g)	T_m_ (°C)	ΔH_m_ (J/g)	X_c_ (%)
P	59.1	109.4	23.8	148.3/157.4	26.3	2.8
P2CN*	55.2	108.8	23.3	147.9/155.8	24.5	1.3
P10low	36.1	83.8	21.3	153.7	32.6	12.1
P10low2CN	32.7	79.8	17.1	152.1	29.2	13.0
P10high2CN	31.4	80.5	17.5	154.2	29.3	12.7
P1low2CN	55.2	104.6	22.2	147.2/156.7	30.2	8.3
P5low2CN	45.3	92.4	24.6	141.4/157.4	27.2	2.7
P10low5CN	36.3	78.8	19.1	153.6	31.1	12.8
P10low12CN	29.8	73.3	20.4	151.6	28.8	9.0

T_g_ = glass transition temperature; T_cc_ = crystallization during heating peak temperature; ΔHcc = enthalpy of crystallization during heating; T_m_ = melting peak temperature; ΔHm = integral of the melting peak.
